# SARCOPENIA, OBESITY AND SARCOPENIC OBESITY IN LIVER TRANSPLANTATION: A BODY COMPOSITION PROSPECTIVE STUDY

**DOI:** 10.1590/0102-672020190001e1434

**Published:** 2019-04-29

**Authors:** Lucilene Rezende ANASTÁCIO, Lívia Garcia FERREIRA, Helem Sena RIBEIRO, Kiara Gonçalves Dias DINIZ, Agnaldo Soares LIMA, Maria Isabel T.D. CORREIA, Eduardo Garcia VILELA

**Affiliations:** 1 Food Science Post-Graduation Program, Universidade Federal de Minas Gerais, Belo Horizonte, MG;; 2 Nutrition and Health Post-Graduation Program, Universidade Federal de Lavras, Lavras, MG;; 3 Instituto de Ensino e Pesquisa, Santa Casa de Belo Horizonte, Belo Horizonte,MG;; 4 Adult Health Post-Graduation Program, Faculty of Medicine, Universidade Federal de Minas Gerais, Belo Horizonte, MG;; 5 Surgery Post-Graduation Program, Faculty of Medicine, Universidade Federal de Minas Gerais, Belo Horizonte, MG Brazil

**Keywords:** Body mass index, Body composition, Liver transplantation, Sarcopenia, índice de massa corporal, Composição corporal, Transplante hepático, Sarcopenia

## Abstract

***Background*:**

Sarcopenia is prevalent before liver transplantation, and it is considered to be a risk factor for morbidity/mortality. After liver transplantation, some authors suggest that sarcopenia remains, and as patients gain weight as fat, they reach sarcopenic obesity status.

***Aim*:**

Prospectively to assess changes in body composition, prevalence and associated factors with respect to sarcopenia, obesity and sarcopenic obesity after transplantation.

***Methods*:**

Patients were evaluated at two different times for body composition, 4.0±3.2y and 7.6±3.1y after transplantation. Body composition data were obtained using bioelectrical impedance. The fat-free mass index and fat mass index were calculated, and the patients were classified into the following categories: sarcopenic; obesity; sarcopenic obesity.

***Results*:**

A total of 100 patients were evaluated (52.6±13.3years; 57.0% male). The fat-free mass index decreased (17.9±2.5 to 17.5±3.5 kg/m^2^), fat mass index increased (8.5±3.5 to 9.0±4.0; p<0.05), prevalence of sarcopenia (19.0 to 22.0%), obesity (32.0 to 37.0%) and sarcopenic obesity (0 to 2.0%) also increased, although not significantly. The female gender was associated with sarcopenia.

***Conclusion*:**

The fat increased over the years after surgery and the lean mass decreased, although not significantly. Sarcopenia and obesity were present after transplantation; however, sarcopenic obesity was not a reality observed in these patients.

## RESUMO

**Racional:**

A sarcopenia é prevalente antes do transplante de fígado e é considerada fator de risco para morbidade/mortalidade desses pacientes. Após o transplante hepático, alguns autores sugerem que a sarcopenia permanece, e os pacientes ganham peso na forma de gordura, atingindo o status de obesidade sarcopênica.

**Objetivo:**

Avaliar prospectivamente as mudanças na composição corporal, prevalência e fatores associados em relação à sarcopenia, obesidade e obesidade sarcopênica após o transplante.

**Métodos:**

Os pacientes foram avaliados em dois momentos diferentes para composição corporal, 4,0±3,2 e 7,6±3,1 anos e após o transplante. Os dados da composição corporal foram obtidos por meio de bioimpedância elétrica. O índice de massa livre de gordura e o índice de massa gorda foram calculados, e os pacientes foram classificados nas seguintes categorias: sarcopênico; obesidade; obesidade sarcopênica.

**Resultados:**

Foram avaliados 100 pacientes (52,6±13,3 anos; 57,0% homens). A prevalência de sarcopenia (19,0% para 22,0%), obesidade (32,0% para 37,0%) e índice de massa livre de gordura (17,9±2,5 para 17,5±3,5 kg/m^2^), índice de massa gorda aumentou (8,5±3,5 para 9,0±4,0 kg/m^2^), e obesidade sarcopênica (0 para 2,0%) também aumentaram, embora não significativamente. O gênero feminino foi associado à sarcopenia.

**Conclusão:**

Após a operação, a gordura aumentou ao longo dos anos e a massa magra diminuiu, embora não significativamente. A sarcopenia e a obesidade estavam presentes após o transplante; no entanto, a obesidade sarcopênica não foi realidade observada nesses pacientes.

## INTRODUCTION

Sarcopenia is a syndrome that is characterized by progressive and generalized loss of skeletal muscle mass and strength associated with adverse outcomes such as physical disability, poor quality of life and death. There are several mechanisms that could be involved in the onset and progression of sarcopenia. They involve, among others, decreased protein synthesis, proteolysis, alterations in neuromuscular integrity and muscle fat content. In an individual with sarcopenia, several mechanisms could be involved, which could vary over time. Recognizing these mechanisms and their underlying causes is expected to facilitate the design of intervention trials that target one or more underlying causes of sarcopenia^9^ ^,^ ^23^. 

The diagnosis of low muscle mass could be accomplished using dual energy X-ray absorptiometry, computed tomography, bioelectrical impedance and sex-specific cutoffs^9^ ^,^ ^31^. Bioelectrical impedance is an easy, fast, low-cost and portable assessment device that can be used to diagnose sarcopenia among post-liver transplant patients^19^ ^,^ ^24^.

Recently, some publications have addressed the sarcopenia diagnosis as well as its outcomes before and after liver transplantation^8^ ^,^ ^28^ ^,^ ^29^. Before the transplant, sarcopenia is highly prevalent, and it is seen in 41-68% of these patients^13^ ^,^ ^20^ ^,^ ^21^ ^,^ ^27^. After surgery, some authors depicted that the prevalence decreases - from 55% before liver transplantation to 30% after^3^, and others have shown that in fact it increases from 62% before liver transplantation (LTx) to 87% after surgery^28^. 

Despite the controversy on what occurs with sarcopenia after LTx, it is well known that patients gain too much weight and become obese after surgery. The median weight gain in the three years after the operation is 11.6±8.7 kg^1^, and the majority of the patients after LTx are overweight or obese^2^. Thus, one may wonder if the weight gain after LTx is a reflection of sarcopenic obesity and not necessarily an improvement in nutritional health^5^. Changes in the body composition of transplant recipients are characterized by an early and inappropriate gain in the fat mass, while the restoration of the body cell mass appears to occur more slowly, and it is incomplete^10^ ^-^ ^26^, which cannot be assessed by BMI (body mass index). 

Therefore, the aim of this study was to verify prospectively the changes in the body composition as well as to depict the prevalence and associated factors of sarcopenia, obesity and sarcopenic obesity in LTx recipients and compare them with BMI status classifications. 

## METHODS

This is a prospective study that involves consecutive long-term liver transplant recipients. For body composition, these patients were evaluated at two different times throughout a four-year span. At the first evaluation, they were 4.0±3.2 years after the liver transplant (median: 3 years; range 0-13 years), and at the second, they were 7.6±3.0 years after the operation (median: 7 years: range: 3-17 years). The study was conducted at a single center (Transplant Outpatient Clinic), and was approved by the Ethics Committee (protocol number 44/08). All patients agreed to participate and signed the written consent. 

The body composition data of the patients were obtained using bioelectrical impedance (RJL Systems^®^ Quantum X, Clinton Township, MI, USA). The previous preparation of the subjects, proposed by the study developer, consisted of the following protocol: subjects should not have exercised or taken a sauna within 8 h of the test and should refrain from alcohol intake for 12 h prior to the test, the height and weight of the subject should be accurately measured and recorded, and the subject should not be moist from sweat or lotion, have a fever, or be in shock. The subjects were also asked to remove metal objects, such as watches, glasses, jewelry, and other objects, which could interfere with the passage of electrical current, and to lie in a supine position with legs apart and arms away in parallel along the body. The electrodes for the bioimpedance measurement were placed on the right side of the body, and they were positioned in pairs on the back of the hand and foot.

The fat-free mass index (FFMI) and fat mass index (FMI) (kg/m^2^) were calculated, and the patients were classified in groups according to the following combinations: sarcopenia (low FFMI and normal FMI); obesity (normal FFMI and high FMI); sarcopenic obesity (low FFMI and high FMI); and normal body composition (normal FFMI and FMI). The FFMI was considered to be low when it was below 17.4 kg/m^2^ in men and below 15.0 kg/m^2^ in women^12^ ^,^ ^17^. The FMI was considered to be high when it was greater than 8.3 kg/m^2^ in men and 11.8 kg/m^2^ in women, according to Kyle et al.^17^ and Gonzalez et al.^12^. The patients were also classified using BMI classifications by the World Health Organization, namely, underweight (BMI<18.5 kg/m^2^), normal (BMI between 18.5-24.9 kg/m^2^), overweight (BMI between 25.0-29.9 kg/m^2^) and obese (BMI>30.0 kg/m^2^)^30^. The data collection included the gender, age, weight, height, and time since the operations, the first weight after LTx (to calculate the weight gain since the operation) and indication for LTx.

### Statistical analysis

The data were evaluated by the Statistical Package for Social Sciences version 17.0 (SPSS Inc., Chicago, IL, USA). The numerical variables underwent the analysis of normality test (Kolmogorov-Smirnov) and presented as the mean and standard deviation because all of them had a normal distribution. Categorical variables were presented as absolute numbers and percentages. Comparisons of body composition data were performed using the paired t test and the Mc Nemar test. Associated factors for sarcopenia, obesity and sarcopenic obesity were assessed using the Chi-Square and Student T test. p values lower than 0.05 were considered to be statistically significant. 

## RESULTS

A total of 100 patients transplanted were evaluated (52.6±13.3 years; 57.0% male). The most frequent indications for LTx were hepatitis C virus cirrhosis (29.0%; n=29), alcohol cirrhosis (26.0%; n=26), autoimmune hepatitis (13.0%; n=13), cryptogenic cirrhosis (11.0%; n=11); hepatocarcinoma (6.0%; n=6) and other indications (28.0%; n=28). 

It was observed that the amount of fat mass and the fat mass index significantly increased, while the percentage of fat-free mass decreased (Table 1, p<0.05). The average weight gain was 1.6±7.2 kg; however, the fat-free mass difference was -0.8±8.0 kg, and the fat mass difference was 1.7±6.9 kg.

**TABLE 1:** Fat-free mass, fat mass and body composition classification among long-term liver transplantation recipients over four years

	First evaluation	Second evaluation	p
	Average±standard deviation % (n)	Average±standard deviation % (n)	
Weight	72.2±16.6	73.9±17.6	0.027*
Body mass index (kg/m^2^)	26.5±5.0	26.3±5.6	0.543
Variables related to fat-free mass			
Fat-free mass (%)	68.4±8.8	66.0±9.9	0.027*
Fat-free mass (kg)	48.2±11.6	48.2±11.6	0.543
Fat-free mass index (kg/m^2^)	17.9±2.5	17.5±3.5	0.162
Total body water (L)	36.3±8.1	36.0±8.3	0.432
Variables related to fat mass			
Fat mass (%)	30.1±10.4	32.1±10.4	0.056
Fat mass (kg)	22.4±9.5	24.2±10.9	0.006*
Fat mass index (kg/m^2^)	8.5±3.5	9.0±4.0	0.038*
Body composition classification			
Sarcopenia	19.0% (19)	22.0% (22)	0.581
Obesity	32.0% (32)	37.0% (37)	0.267
Sarcopenic obesity	0.0% (0)	2.0% (2)	-
Normal	49.0% (49)	39.0% (39)	0.263

Paired t test and Mc Nemar; *=p<0.05

The prevalence of sarcopenia, obesity and sarcopenic obesity also increased over the years, but not significantly. Sarcopenia was prevalent in 22.0% (n=22) and obesity in 37.0% (n=37) of the patients over the long term after LTx. These disorders were widely prevalent on both assessed occasions, with only 49% of the patients having a normal body composition in the first assessment. However, sarcopenia and obesity do not co-exist in liver transplant recipients, as none in the first assessment and only two patients (in the later) were diagnosed with sarcopenic obesity (Table 1).

**FIGURE 1:**
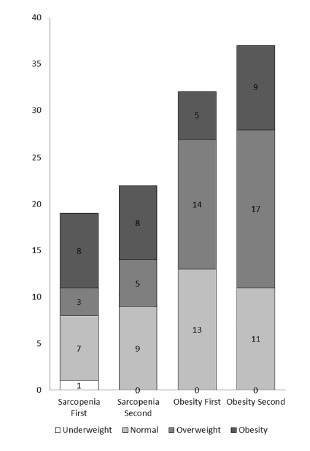
Nutritional status classification using body mass index among liver recipients classified as sarcopenic and obese over four years

Considering the nutritional status by BMI, four patients were underweight at the first assessment and two at the latter. Overweight was observed in 38 patients in the first evaluation and in 31 in the second, while the obesity rose from 22 to 27 patients. It was observed that sarcopenic patients were predominantly normal, overweight or obese according the BMI. Only one patient was underweight and sarcopenic in the first evaluation, and none in the second. Overweight and obesity by BMI were also considered in obese patients by body composition analysis; however, a substantial portion of them presented with normal BMI and were classified as obese by body composition assessment (Figure 1). Only two patients who were classified as sarcopenic obese were overweight and obese by BMI at the first assessment. Sarcopenic patients had similar BMIs to non-sarcopenic patients in both evaluations (Table 2), and in addition, with regard to the obese patients, the obese and non-obese patients by body composition did not differ with respect to BMI (Table 3).

**TABLE 2:** Variable distribution among sarcopenic liver transplantation recipients over four years

Evaluation	First evaluation			Second evaluation		
	Non-sarcopenic	Sarcopenic	p	Non-sarcopenic	Sarcopenic	p
	(n=81)	(n=19)		(n=78)	(n=22)	
Age (years)	45.2±13.2	41.5±14.9	0.286	53.1±13.2	50.8±13.9	0.489
Time since LTx (years)	4.0±3.2	4.1±3.2	0.972	7.7±3.2	7.1±2.4	0.421
BMI (kg/m^2^)	25.7±4.3	28.7±8.9	0.316	26.3±4.8	27.6±5.7	0.286
Weight gain since LTx	10.0±8.8	11.0±8.9	0.671	12.1±11.3	11.4±8.9	0.788
Sex						
Males	87.7%(50)	12.3%(7)	0,049*	86.0%(49)	14.0%(8)	0.027*
Females	72.1%(31)	27.9%(12)		67.4%(29)	32.6%(14)	
Indication for transplant						
Hepatitis C virus	79.3%(23)	20.7%(6)	0.783	79.3%(23)	20.7%(6)	0.840
Ethanolic cirrhosis	80.8%(21)	19.2%(19)	0.972	84.65(22)	15.4%(4)	0.344
Hepatitis auto-immune	92.35(12)	7.7%(1)	0.265	84.6%(11)	15.4%(2)	0.537
Cryptogenic cirrhosis	81.0%(81)	19.05(19)	0.942	81.8%(9)	18.2%(2)	0.746
Hepatocarcinoma	100.05(6)	0.0%(0)	0.221	83.3%(5)	16.75(1)	0.605
Others	78.6%(22)	21.45(6)	0.699	71.4%(20)	28.6%(8)	0.323

BMI=body mass index; LTx=liver transplantation; Chi-square and T student test; *=p<0.05

**TABLE 3:** Variable distribution among obese liver transplantation recipients over four years

Evaluation	First evaluation			Second evaluation		
	Non-obese	Obese	p	Non-obese	Obese	p
	(n=68)	(n=31)	value	(n=63)	(n=37)	
Age (years)	45.0±13.3	43.5±14.1	0.607	52.6±13.2	52.5±13.8	0.974
Time since LTx (years)	3.9±3.2	4.3±3.2	0.634	7.2±2.7	8.2±3.4	0.090
BMI (kg/m^2^)	26.4±6.1	26.0±4.3	0.752	26.3±5.5	26.9±4.1	0.591
Weight gain since LTx	10.4±9.2	9.6±8.0	0.656	12.1±10.7	11.7±11.1	0.871
Sex						
Males	63.2%(36)	36.8%(21)	0.232	56.1%(32)	43.9%(25)	0.102
Females	74.4%(32)	25.6%(11)		72.1%(31)	27.9%(12)	
Indication for transplant						
Hepatitis C virus	65.5%(19)	34.5%(10)	0.734	65.5%(19)	34.5%(10)	0.739
Ethanolic cirrhosis	61.5%(16)	38.5%(10)	0.412	50.0%(13)	50.0%(13)	0.110
Hepatitis auto-immune	69.2%(9)	30.8%(4)	0.919	53.8%(7)	46.2%(6)	0.464
Cryptogenic cirrhosis	81.8%(9)	18.2%(2)	0.298	81.8%(9)	18.2%(2)	0.171
Hepatocarcinoma	50.0%(3)	50.0%(3)	0.330	66.7%(4)	33.3%(2)	0.848
Others	67.9%(19)	32.1%(32)	0.985	67.9%(19)	32.1%(9)	0.530

BMI=body mass index; LTx = liver transplantation; Chi-square and T student test; *=p<0.05.

Sarcopenia and obesity were not affected by age, time since transplantation, weight gain since transplant, and indication for liver transplant. Sarcopenic patients were predominantly women in both evaluations (12 of 19 patients in the first evaluation and 14 of 22 patients in the last evaluation) (Table 2). Obesity was similar among women and men (Table 3).

## DISCUSSION

Sarcopenia is frequently associated with wasting syndromes and chronic diseases, such as cirrhosis. The presence as well as the consequences of this condition are well described in liver pre-transplant patients^8^ ^,^ ^16^ ^,^ ^26^ ^,^ ^29^. However, there are few data in the literature that report the prevalence, causes and impact of sarcopenia and sarcopenic obesity in patients after liver transplantation. Furthermore, when these data are reported, there are different methods and cutoff points to identify the prevalence, which hinders a comparison of the results obtained with this specific population.

In the present study, sarcopenia, obesity and sarcopenic obesity, as defined by body composition, increased over the four years, but not significantly. The prevalence of sarcopenia increased from 19% to 22% and obesity from 32% to 37%. Sarcopenic obesity was not observed in patients at the first time point measured, but two of them acquired this condition four years later. 

The prevalence of sarcopenia in long-term LTx patients is lower than that reported by other authors. Bergerson et al.^3^ conducted a study with 40 patients (alcoholic cirrhosis, non-alcoholic steatohepatitis cirrhosis, and primary sclerosing cholangitis cirrhosis) using images of computed tomography, and the reported the prevalence of sarcopenia in 55% pre-LTx patients, which decreased to 30% after transplantation (12-48 months after LTx). They considered skeletal muscle index cutoff levels for sarcopenia of <38.5 cm^2^/m^2^ in women and <52.4 cm^2^/m^2^ in men. A higher prevalence of sarcopenia was found by Tsien et al.^28^ by using images of computed tomography in addition to other measurements, and they reported that sarcopenia increased from 62.3% of their patients pre-transplant to 87% post-transplant, 19.3±9 months after liver transplant. Additionally, in these patients, the fat area increased in 23 (43.4%) and remained unchanged or decreased in the remaining 30 (56.6%). In contrast to the previous reports on the development of obesity and the increase in fat mass after transplantation, the mean visceral and subcutaneous fat mass were unaltered in this cohort after transplantation^28^. Another study accessed 42 patients after liver transplantation, and of these, 48% had mid-arm fat area values that were above the 90^th^ percentile of the normal population, and they had a significantly lower body cell mass (BCM) (measured by the phase angle and compared with healthy controls), which reflects an abnormal body composition^26^. These authors concluded that the changes are characterized by an early and inappropriate gain in the fat mass, while the restoration of the BCM appears to occur more slowly and incompletely. The differences among the percentages of sarcopenia in the present study compared to other studies could be due to (in addition to the different methods and cutoffs) the longer time for the post-transplant assessment of the patients (an average of 4.0±3.2 years after transplant in the first evaluation and 7.6±3.0 years in the last evaluation). Some of the authors suggest that sarcopenia does not progress but is arrested and frequently improves after surgery^3^ and that sarcopenia progresses after LTx initially and does not recover at least within the first year after surgery^6^.

There are limited data about sarcopenic obesity in liver transplantation recipients, although weight gain and obesity are well established after transplantation^1^ ^,^ ^7^ ^,^ ^26^. Choudhary et al.^7^ evaluated 82 patients 24 months after LTx (varying from 12 to 38.5 months) by bioelectrical impedance. Although the cutoffs for sarcopenia were not described and obesity was defined by BMI>25 kg/m^2^ plus visceral obesity, 88% of the patients were identified with sarcopenic obesity. In our study, BMI>25 kg/m^2^ was seen in 58% of the patients in the first evaluation, but sarcopenic obesity according to the definition of Kyle et al.^17^ and Gonzalez et al.^12^ was seen in only 2%. The latter study evaluated the presence of sarcopenia in 175 cancer patients who were assessed before chemotherapy, and when both FMI and FFMI cutoffs were used, 58% of the patients were classified as obese, while only 1% were classified as presenting with a concurrent high FMI and low FFMI (sarcopenic obese)^12^.

In the present study, sarcopenic patients presented mostly as normal, overweight or obese by BMI. In addition, many patients with obesity by FMI were considered to be normal by the BMI classification. BMI does not accurately differentiate between lean from fat tissues, and thus, it does not provide information on whether excess FM, excess lean mass, or excess of both masses are present in patients who have excessive weight by the BMI criteria^12^. Furthermore, a normal or excessive weight for height does not necessarily mean lean mass because sarcopenic patients can be found among them. Additionally, patients with and without sarcopenia had similar BMIs, and the same held for obese patients who were identified by body composition. Obese patients also had similar weight gain since LTx than those without obesity.

Conversely, Jeon et al.^25^ showed a decrease in the BMI over the years (24.6±3 kg/m^2^ pre-liver transplant to 23.3±3.1 kg/m^2^ post-transplant, p=0.015) in sarcopenic patients compared to those without sarcopenia. Most of these patients (79%, n=52, p<0.001) already had sarcopenia before liver transplantation, and an increase was observed in the incidence of sarcopenia after liver transplantation. Another study that enrolled patients on the waiting list for liver transplantation showed that their BMI was associated with an increased prevalence of sarcopenia [100% (8/8) with a BMI<18.5 kg/m^2^ (underweight), 46% (29/63) with a BMI 18.6-25 kg/m^2^ (normal weight), and 30% (21/71) with a BMI>25 kg/m^2^ (overweight), p<0.001]. The mean BMI of the sarcopenic patients was within the normal weight range, which is the same result that was found in our study (24.2 kg/m^2^ - IQR ¼ 20.4-26.2 kg/m^2^) ^27^.

The only associated factor that was identified as a risk factor for sarcopenia was gender, because age, prior obesity, and indication for transplant did not have an impact. Surprisingly, the prevalence of sarcopenia was significantly higher in women (27.9% in the first assessment and 32.6% in the last) than in men (12.3% and 14%), which differs from studies that showed the opposite^4^ ^,^ ^21^. In aging men, the percentage of fat mass increases initially, and then it levels off or decreases. Such a change has been attributed to an accelerated decrease in the lean mass, along with an initial increase and a later decrease in the fat mass. Women show a generally similar pattern. The intramuscular and visceral fat increased with age while the subcutaneous fat declined^9^.

The lack of unanimous criteria to define low muscle mass and high fat mass to identify cases of sarcopenic obesity represents a major clinical and research drawback^24^. Different methods and cutoffs make it difficult to compare data. Additionally, a consensus on sarcopenia^9^ ^,^ ^23^ recommends that the diagnosis could be made in the presence of both low muscle mass and low muscle function (strength or performance), but most studies that were conducted on LTx patients used only low muscle mass. The criteria are somewhat arbitrary and study-specific, which could have minimized the predictive value of sarcopenic obesity as a health risk factor^25^. The lack of standardized diagnostic approaches is reflected in the variable combination of body composition indices and cutoffs that have been used to classify sarcopenic obesity. The latter could be a limitation of the risk prediction when considering either sarcopenia or obesity alone^24^. In a study with a general middle-age population (n=654), the prevalence of sarcopenia ranged from 0% to 45.2%, depending on the used methods and cutoffs, and only one participant was identified as having sarcopenia according to all of the diagnostic criteria^4^. The gold standard assessments the body composition involve body-imaging techniques, such as computed tomography and magnetic resonance followed by dual energy X-ray absorptiometry (DXA), as the preferred alternative method. 

There are some limitations with regard to the use of bioelectrical impedance, as differences in the measurement protocols can be found, resulting in proportional or positional bias. In addition, the use of statistically derived, population-specific equations (typically height, weight, age, gender, and ethnicity specific) that have mostly been validated among healthy and normal-weight individuals under highly controlled conditions^22^ and cross-validation in independent samples is generally lacking, except among healthy adults^18^. In addition, assessments of change in the FFM with single-frequency bioimpedance, especially after weight loss or gain either intentionally or owing to illness, could differ from reference measurements^18^. Nevertheless, in clinical practice, the European Working Group on Sarcopenia in Older People^9^ indicates bioelectrical impedance analysis (BIA) as a good portable alternative method. BIA has also been suggested for the systematic and repeated evaluation of FFM in clinical practice because it is safe, portable and inexpensive, and in addition, it is quick and simple to use^14^. BIA measurement techniques, when used under standard conditions, have been studied for >10 years and may be a good alternative to DXA. The measurements are sensitive to subject conditions such as hydration and recent activity, and the instrument predictions could be population specific. However, a study was performed with 215 teenagers to assess the predictive ability of four different BIA devices with and without a performance protocol. When compared to DXA, it was concluded that in most cases, the BIA data were similar to DXA in the same evaluation without protocol, which indicates the reliability of data when it is not possible to perform the protocol^11^. Indicators of fat free mass and fat mass have been adjusted to account for the differences in body sizes. The adjustment for height squared has been the preferred method, but the adjustment for body weight has also been utilized^24^. Some limitations of the study should be highlighted, as the lack of an initial evaluation prior to transplantation or immediately after surgery to know the baseline situation of the patients and be able to study their subsequent evolution.

## CONCLUSION

Fat mass increased over the years after surgery, and lean mass decreased, although not significantly. Sarcopenia and obesity were highly present after LTx, with increasing prevalence over the years, which resulted in less than half of the patients having a normal body composition at both evaluations. However, sarcopenic obesity was not a reality that was observed in these long-term patients. The BMI, weight gain, age and indication for LTx did not affect sarcopenia and obesity. Females had a greater prevalence of sarcopenia. Sarcopenic patients were the majority among normal, overweight or obese patients by BMI, and some obese patients by BIA were classified as normal weight by BMI criteria.
